# Urethral Sphincter Length but Not Prostatic Apex Shape in Preoperative MRI Is Associated with Mid-Term Continence Rates after Radical Prostatectomy

**DOI:** 10.3390/diagnostics12030701

**Published:** 2022-03-13

**Authors:** Benedikt Hoeh, Mike Wenzel, Matthias Müller, Clarissa Wittler, Eva Schlenke, Jan L. Hohenhorst, Jens Köllermann, Thomas Steuber, Markus Graefen, Derya Tilki, Simon Bernatz, Pierre I. Karakiewicz, Felix Preisser, Andreas Becker, Luis A. Kluth, Philipp Mandel, Felix K. H. Chun

**Affiliations:** 1 Department of Urology, University Hospital Frankfurt, Goethe University Frankfurt am Main, 60596 Frankfurt am Main, Germany; mikewenzel91@aol.com (M.W.); matthias.mueller@kgu.de (M.M.); clarissa.wittler@kgu.de (C.W.); eva.schlenke@gmx.de (E.S.); felix.preisser@kgu.de (F.P.); andreas.becker@kgu.de (A.B.); luis.kluth@kgu.de (L.A.K.); philipp.mandel@kgu.de (P.M.); felix.chun@kgu.de (F.K.H.C.); 2 Cancer Prognostics and Health Outcomes Unit, Division of Urology, University of Montréal Health Center, Montréal, QC H4A 3J1, Canada; lukas.hohenhorst@gmail.com (J.L.H.); pierrekarakiewicz@gmail.com (P.I.K.); 3 Martini-Klinik Prostate Cancer Center, University Hospital Hamburg-Eppendorf, 20246 Hamburg, Germany; steuber@uke.de (T.S.); graefen@uke.de (M.G.); dtilki@me.com (D.T.); 4 Dr. Senckenberg Institute of Pathology, University Hospital Frankfurt, 60323 Frankfurt am Main, Germany; jens.koellermann@kgu.de; 5 Department of Urology, University Hospital Hamburg-Eppendorf, 20246 Hamburg, Germany; 6 Department of Urology, Koc University Hospital, Istanbul 34450, Turkey; 7 Department of Radiology, University Hospital Frankfurt, 60323 Frankfurt, Germany; simon.bernatz@kgu.de

**Keywords:** radical prostatectomy, mid-term urinary continence, functional outcome, prostate cancer, Lee-type, apex, membranous urethra

## Abstract

Background: To test the impact of urethral sphincter length (USL) and anatomic variants of prostatic apex (Lee-type classification) in preoperative multiparametric magnet resonance imaging (mpMRI) on mid-term continence in prostate cancer patients treated with radical prostatectomy (RP). Methods: We relied on an institutional tertiary-care database to identify patients who underwent RP between 03/2018 and 12/2019 with preoperative mpMRI and data available on mid-term (>6 months post-surgery) urinary continence, defined as usage 0/1 (-safety) pad/24 h. Univariable and multivariable logistic regression models were fitted to test for predictor status of USL and prostatic apex variants, defined in mpMRI measurements. Results: Of 68 eligible patients, rate of mid-term urinary continence was 81% (n = 55). Median coronal (15.1 vs. 12.5 mm) and sagittal (15.4 vs. 11.1 mm) USL were longer in patients reporting urinary continence in mid-term follow-up (both *p* < 0.01). No difference was recorded for prostatic apex variants distribution (Lee-type) between continent vs. incontinent patients (*p* = 0.4). In separate multivariable logistic regression models, coronal (odds ratio (OR): 1.35) and sagittal (OR: 1.67) USL, but not Lee-type, were independent predictors for mid-term continence. Conclusion: USL, but not apex anatomy, in preoperative mpMRI was associated with higher rates of urinary continence at mid-term follow-up.

## 1. Introduction

Urinary incontinence after radical prostatectomy (RP) has consistently been reported as a bothersome complication for prostate cancer (PCa) patients and is associated with a substantial loss of quality of life in affected patients [1,2,3,4]. Besides different patient characteristics that have been postulated as potential risk factors for post-RP urinary incontinence, anatomical features based on preoperative multiparametric magnetic resonance imaging (mpMRI) have been suggested to be associated with urinary continence [5,6,7,8]. Since mpMRI has found its way to current guidelines as a fundamental pillar in prostate cancer diagnostics, localization, and staging, the ability to identify patients at higher risk of developing urinary incontinence, relying on mpMRI, would be of crucial importance to offer those patients intensified postoperative management to minimize the impact of quality of life caused by urinary incontinence [7,9]. Hereby, prostatic apex variations as well as urethral sphincter length have been reported to be of predictive nature for urinary incontinence after RP. Recently, Wenzel et al. reported that apex Lee-type C and D, as well as urethral sphincter length, were associated with higher rates of very early urinary continence [10]. However, the authors did not investigate the effect of mpMRI findings on urinary continence at a longer follow-up [10]. Even though previous studies have investigated the association between prostatic apex variations and length of urethral sphincter on urinary continence, the results were either based on a historical cohort or early follow-up time period, or they relied on a North American/Asian study cohort [11,12,13,14,15]. We hypothesized that prostatic apex variants as well as urethral sphincter length represent independent predictors for mid-term urinary continence, in line with previous findings by Wenzel et al. for very early urinary continence [10]. To address this void, we relied on a contemporary cohort of PCa patients treated with RP.

## 2. Material and Methods

### 2.1. Study Population

From 03/2018 to 12/2019, PCa patients treated with RP (n = 321) at the Department of Urology at the University Hospital Frankfurt, Germany, were retrospectively identified from our prospective institutional database. Patients without mpMRI prior to RP (n = 194) or missing information regarding continence status following RP (n = 59) were excluded from further analyses. Those criteria resulted in 68 patients, who represented the final study cohort. Indication for RP was biopsy-confirmed prostate cancer. All surgeons who performed RP in the current cohort were experienced surgeons trained in high-volume prostate cancer centers. RP was routinely performed with full functional-length urethral sphincter (FFLU) and neurovascular bundle preservation (NVBP) with intraoperative frozen section technique (IFT), as previously described [16].

### 2.2. MpMRI: Lee-Type Definition and Urethral Sphincter Length

**Figure 1:**
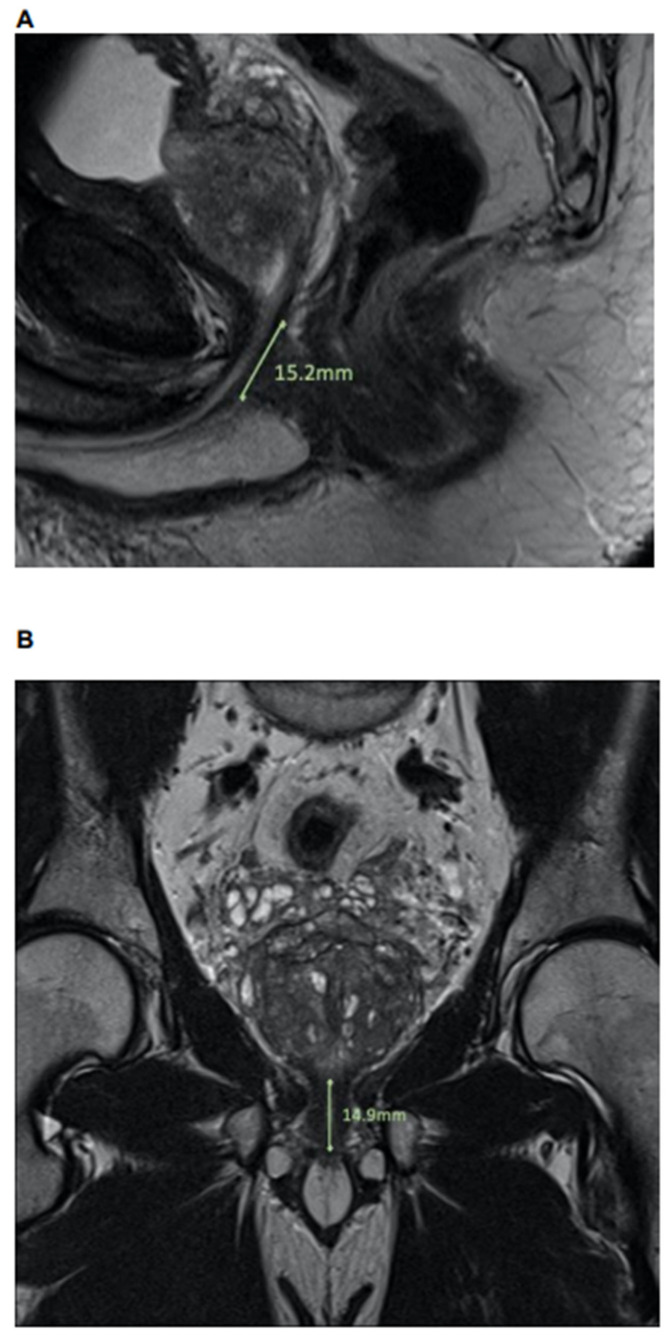
Urethral sphincter length measurements in (**A**) sagittal and (**B**) coronal dimensions in a multiparametric MRI prior to radical prostatectomy.

MpMRI were performed as recommended by the European Society of Urogenital Radiology (ESUR) and previously described [17]. Relying on T2-weighted sequences in mpMRI, anatomical variants of prostatic apex were classified relying on the four-tier classification system by Lee et al. [5]. Here, prostatic apex was categorized as (Lee-type) A, B, C, and D. Lee-type A was defined as a prostatic apex overlapping the membranous urethra anteriorly, as well as posteriorly. Lee-type B and C were classified as an overlap of the prostatic apex of the anterior or posterior membranous urethra, respectively. Finally, Lee-type D was defined as lack of overlap of the prostatic apex over the membranous urethra in mpMRI [5]. Second, urethral sphincter length and diameter were measured (in millimeters (mm)) in sagittal, coronal, and axial directions in preoperative mpMRI, as previously described (Figure 1) [10]. MpMRI analyses were performed by a specialist in urologic imaging, supervised by a board-certificated radiologist, in a blinded fashion without the knowledge of endpoint of interest.

### 2.3. Outcome Measurements

Mid-term urinary continence (>6 months post-surgery) was defined as the use of no or one safety pad within 24 h, whereas a higher number of pads was considered incontinent. Data on urinary continence status was extracted of voluntary self-reported standardized, validated questionnaires.

### 2.4. Statistical Analyses

Descriptive statistics included frequencies and proportions for categorical variables. Medians and interquartile ranges (IQR) were reported for continuously coded variables. The chi-squared test examined the statistical significance of the differences in proportions, while the Kruskal–Wallis test was used to examine differences in medians. Statistical analyses consisted of two parts. In the first part, tabulation of the overall cohort according to (a) surgical approach and (b) urinary continence status was performed. In the second part of the analyses, three separate sets of univariable and multivariable logistic regression models were fitted to test the relationship between (a) urethral sphincter length coronal, (b) urethral sphincter length sagittal, and (c) prostatic apex Lee-type and urinary continence following RP. Specifically, logistic regression models were additionally set for the covariables of age, prostate volume, pT-stage (pT2 vs. >pT2), surgical approach (open vs. robotic-assisted RP), nerve sparing approach (none vs. yes), and pathological International Society of Urological Pathology (ISUP) Score (1/2/3 vs. 4/5). Covariables, which were statistically significant in univariable logistic regression models, were used for adjustment in multivariable models. For all statistical analyses, the R software environment for statistical computing and graphics (version 3.4.3) was used [18]. All tests were two-sided with a level of significance set at *p* < 0.05.

## 3. Results

### 3.1. Descriptive Characteristics of the Study Population

Between 03/2018 and 12/2019, 68 patients underwent RP with data available for mpMRI prior to RP and mid-term urinary continence status. Of those, 37 (55%) underwent robotic-assisted and 31 (45%) open RP (Table 1). Median age was 66 years (interquartile range (IQR): 58–72), prostate-specific antigen was 7 ng/mL (IQR: 5–10), and median prostate volume was 35 mL (IQR 28–45) in the overall cohort. Unfavorable characteristics, such as high-risk D’Amico score and ISUP 4/5 at biopsy were present in 22 (33%) and 18 (27%) patients in the overall cohort, respectively (Table 1). Final histopathological examination showed in 27 patients (40%) >pT2-stage and in 3 patients (4%) positive lymph-node invasion (Table 1). Additional stratification was performed according to mid-term continence status (Table 2).

### 3.2. Continence Rates and Urethral Sphincter Length and Lee-Type in mpMRI

At mid-term follow-up, urinary continence rate was 81% (n = 55; Table 2). Median lengths of the urethral sphincter were 14.7 mm (IQR: 13.0–16.7), 15.1 mm (IQR: 12.8–16.8), and 10.2 mm (IQR: 9.2–11.2) in coronal, sagittal, and axial directions, respectively. Median diameter of urethral sphincter was 9.1 mm (IQR: 8.0–10.1). Moreover, 11 (16%), 5 (7.4%), 5 (7.4%), and 47 (69%) patients exhibited Lee-type A, B, C, and D in preoperative mpMRI, respectively.

Following stratification according to urinary continence status, the median lengths of the urethral sphincter in the coronal (15.1 vs. 12.5 mm; *p* = 0.009) and sagittal (15.4 vs. 11.1 mm; *p* < 0.001) directions were significantly longer in continent vs. incontinent patients (Table 2). No difference in length of the urethral sphincter in the axial direction or the diameter of urethral sphincter was recorded in continent vs. incontinent patients (both *p* = 0.5). Furthermore, Lee-type distributions did not differ between continent vs. incontinent patients. Here, rates for Lee-type A, B, C, and D were 15, 5, 7, and 73% and 23, 15, 8, and 54% for continent and incontinent patients, respectively (*p* = 0.4).

### 3.3. Univariable and Multivariable Logistic Regression Models

In univariable logistic regression models, the length of the urethral sphincter measured in coronal (odds ratio (OR): 1.42; 95%-CI: 1.09–1.96; *p* = 0.02) and sagittal (OR: 1.69; 95%-CI: 1.31–2.33; *p* < 0.001) directions were independent predictors for mid-term continence status in patients treated with RP. Moreover, age (OR: 0.88; 95%-CI: 0.79–0.97; *p* = 0.02), surgical approach (OR: 5.4; 95%-CI: 1.46–26.17; *p* = 0.02), nerve sparing (OR: 11.78; 95%-CI: 2.01–94.71; *p* = 0.01), and pathological ISUP (OR: 0.12; 95%-CI: 0.03–0.51; *p* = 0.004) were independent predictors for urinary continence in univariable logistic regression models (Table 3 and Table 4). 

Specific, separate multivariable logistic regression model testing for independent predictor status for (a) length of urethral sphincter in coronal direction, (b) length of urethral sphincter in sagittal direction, and (c) apex Lee-type exhibited independent predictor status for the length of the urethral sphincter in the coronal direction (OR: 1.35; 95%-CI: 1.01–1.96; *p* = 0.045) and for length of the urethral sphincter in the sagittal direction (OR: 1.67; 95%-CI: 1.22–2.52; *p* = 0.005). Conversely, apex Lee-type failed to reach a statistically significant predictor status in multivariable logistic regression models (*p* ≥ 0.1; Table S1). In all three sets of multivariable logistic regression models, higher age was associated with lower chances of urinary continence (OR range: 0.80–0.87; all *p* < 0.05). 

## 4. Discussion

Previously, Wenzel et al. reported that prostate apex shape of Lee-type C (OR: 1.53) and D (OR: 1.27) in mpMRI provided the best prediction for very early continence in a contemporary cohort of prostate cancer patients treated with RP [10]. Moreover, length of urethral sphincter in the sagittal direction was reported to be a moderate yet statistically significant predictor for very early continence (OR: 1.03) [10]. Unfortunately, later time points, other than very early continence rates, were not investigated by Wenzel et al. [10]. Identification of patients with higher risk of urinary incontinence after RP may be of great importance, since intensified pelvic floor training following RP could be planed a priori and patients could be consulted accordingly. We hypothesized that prostatic apex anatomy as well as length of the urethral sphincter in preoperative mpMRI remain independent predictors in contemporary RP patients at mid-term follow-up for urinary continence. We tested this hypothesis, relying on a contemporary cohort of RP patients from within our institutional database, and made several noteworthy observations. 

First, median urethral sphincter length differed significantly between patients reporting continence at mid-term relative to incontinent patients (*p* < 0.05). Specifically, median length of urethral sphincter was statistically longer, irrespective of if measurements were performed in the coronal (15.1 vs. 12.5 mm) or sagittal (15.4 vs. 11.1 mm) layer (both *p* < 0.05). Conversely, no difference was recorded for urethral sphincter length in the axial direction nor for the urethral sphincter diameter (both *p* ≥ 0.5). The current findings are in agreement with previously reported observations, where continent patients exhibited longer urethral sphincter measurements relative to their non-continent patients [9,10,12,19]. It is of note that the current findings support and contribute to reports indicating that interracial differences in urethral sphincter length may exist [14]. Indeed, the median urethral sphincter length recorded in the current study cohort exceeds previously reported urethral sphincter lengths recorded in Asian prostate cancer patients treated with RP [11,20,21]. Consequently, interpretation and comparison of urethral sphincter length should be performed cautiously since interracial differences are likely to represent a non-negligible bias.

Second, urethral sphincter length remained a profound predictor for urinary continence in multivariable logistic regression models after adjustment for potentially confounding covariates. Interestingly, refitting multivariable logistic regression models for urethral sphincter length relying on measurements in coronal (OR: 1.35) or sagittal (OR: 1.67) directions did not change the profound predictor status of urethral sphincter length on mid-term urinary continence. The current study indicates that measurements of urethral sphincter length are not only restricted to sagittal directions, as reported in the majority of previous reports, but can also be performed in the coronal axis. Since the majority of previous reports either relied on short-term (post-catheter removal–<3 months) or long-term (>12 months) urinary continence rates, a direct comparison to the current findings cannot be performed [8]. Exceptions consisted of four studies that investigated the effect of urethral sphincter length on mid-term (6 months) urinary continence. For example, Sauer et al. reported a moderate positive correlation of urethral sphincter length and urinary continence (OR: 0.8; *p* = 0.01), relying on a cohort of RP treated patients between 2014 and 2018 [22]. Moreover, Mungovan et al., in a systematic review, reported a modest positive effect of urethral sphincter length on urinary continence at 6 months, indicated by an aggregate odds ratio of 1.12 (*p* < 0.001). It is of note that those results were based on only three, more historical studies, of which two were performed on a North American and Asian study population [8,13,23,24]. The current findings underline and surpass findings by previous authors in terms of magnitude. Moreover, the current study demonstrated that urethral sphincter length is a profound, reliable predictor of mid-term urinary continence in a most contemporary cohort of RP patients. 

Third, prostatic apex conformation, categorized according to Lee et al., was not associated with mid-term urinary continence in the current study [5]. Even though the previous authors reported a positive association between Lee-type C and D, those findings were not apparent when mid-term continence status was the primary endpoint [5,10]. The current study complements previous findings, which indicated that apex conformation does not play a crucial role for urinary continence in long-term follow-up [22]. Consequently, it may be postulated that prostatic apex conformation does not affect urinary continence when longer follow-up periods are chosen as primary endpoints. 

Fourth and finally, among all other covariables, age surfaced solely as an independent predictor for mid-term urinary continence. These observations remained unchanged throughout all three separate sets of multivariable regression models. Even though age represented a covariable for adjustment, when mpMRI (urethral sphincter length, Lee-type) findings were investigated, the moderate negative association with age and mid-term urinary continence (OR range: 0.80–0.87; all *p* < 0.05) is in line with previous reports [22]. It is of note that robotic-assisted RP was not statistically significant associated with mid-term continence rates in multivariable analyses (*p* > 0.05).

Despite these noteworthy findings, the current study is not devoid of limitations. First, the study has to be interpreted in the light of its retrospective design and sample size limitations. Second, even though mpMRI were analyzed by an experienced specialist in urological imaging, who was supervised by a board-specific radiologist, interobserver variability cannot formally be ruled out. Third, the current study cohort included both robotic-assisted and open RP patients. We acknowledge that previous studies have postulated benefits of robotic-assisted RP over open RP in the light of postoperative surgical outcomes. Conversely, large-scaled studies, such as those reported by Haese et al., relying on an overall cohort of 10,790 RP patients (open and robotic-assisted), demonstrated no differences for urinary continence rates in 3 and 12 months follow-up between robotic-assisted vs. open RP [25,26]. To address this potential confounding bias, all multivariable analyses were adjusted for the surgical approach. Moreover, similar considerations applied to nerve-sparing. Again, to address for a potential confounding bias, multivariable analyses were additionally adjusted for the nerve sparing approach [27]. Fifth, and finally, a potential bias regarding the extent of postsurgical pelvic floor training cannot be ruled out. It is of note that all patients were strongly encouraged to seek professional pelvic-floor training for urinary continence recovery and were already instructed during their in-patient stay.

## 5. Conclusions

Length of urethral sphincter but not apex anatomy in preoperative mpMRI was associated with higher rates of urinary continence at mid-term follow-up. Specifically, measurement of length of the urethral sphincter in both coronal and sagittal directions exhibited independent predictor status, underlying the reliability of mpMRI evaluation.

Relying on the current findings, patients with higher risk of urinary incontinence following RP may be identified on the basis of mpMRI findings.

## Figures and Tables

**Table 1:** Patient and clinicopathological characteristics of 86 radical prostatectomy patients between 03/2018 and 12/2019, stratified according to surgical approach (robotic-assisted vs. open); all values are median (IQR) or frequencies (%).

	Overall, N = 68 (100%)	Robotic-Assisted RP, N = 37 (55%)	Open RP, N = 31 (45%)	*p*-Value
Length of urethral sphincter, coronal, in mm Median (IQR)	14.7 (13.0, 16.7)	15.0 (13.7, 17.1)	14.5 (12.2, 16.2)	0.2
Length of urethral sphincter, sagital, in mm Median (IQR)	15.1 (12.8, 16.8)	15.1 (14.1, 17.0)	15.3 (10.8, 16.7)	0.4
Length of urethral sphincter, axial, in mm Median (IQR)	10.2 (9.2, 11.2)	9.9 (9.2, 11.1)	10.2 (9.1, 11.2)	0.8
Diameter of urethral sphincter, coronal, in mm Median (IQR)	9.1 (7.9, 10.1)	9.4 (8.2, 10.1)	8.7 (7.6, 9.8)	0.2
Lee-type, n (%)				0.3
A	11 (16%)	5 (14%)	6 (19%)	
B	5 (7%)	1 (3%)	4 (13%)	
C	5 (7%)	4 (11%)	1 (3%)	
D	47 (69%)	27 (73%)	20 (65%)	
Age in years, Median (IQR)	66 (58, 72)	66 (58, 70)	66 (60, 72)	0.6
PSA, in ng/mL, Median (IQR)	7 (5, 10)	6 (5, 8)	8 (6, 15)	0.005
Body-Mass Index in kg/m^2^, Median (IQR)	25.6 (24.0, 27.2)	25.7 (23.9, 27.1)	25.4 (24.2, 27.7)	0.6
Prostate volume in mL, Median (IQR)	35 (28, 45)	35 (29, 44)	36 (28, 49)	0.7
D’Amico Score, n (%)				<0.001
Low	5 (8%)	4 (11%)	1 (3%)	
Intermediate	39 (59%)	28 (78%)	11 (37%)	
High	22 (33%)	4 (11%)	18 (60%)	
ISUP Score at biopsy, n (%)				<0.001
ISUP1	7 (10%)	4 (11%)	3 (10%)	
ISUP2	26 (38%)	21 (57%)	5 (16%)	
ISUP3	17 (25%)	8 (22%)	9 (29%)	
ISUP4	10 (15%)	4 (11%)	6 (19%)	
ISUP5	8 (12%)	0 (0%)	8 (26%)	
Pathological ISUP Score, n (%)				<0.001
ISUP1	8 (12%)	7 (19%)	1 (3%)	
ISUP2	30 (46%)	23 (62%)	7 (26%)	
ISUP3	14 (22%)	5 (14%)	9 (32%)	
ISUP4	2 (3%)	0 (0%)	2 (7%)	
ISUP5	11 (17%)	2 (5%)	9 (32%)	
Nerve sparing approach, n (%)				0.003
None	6 (9%)	0 (0%)	6 (19%)	
Yes	62 (91%)	37 (100%)	25 (81%)	
pT-stage, n (%)				0.02
pT2	41 (60%)	27 (73%)	14 (45%)	
>pT2	27 (40%)	10 (27%)	17 (55%)	
pN-stage, n (%)				0.2
pN0	60 (88%)	34 (92%)	26 (84%)	
pN1	3 (4%)	0 (0%)	3 (10%)	
pNx	5 (8%)	3 (8%)	2 (6%)	

Abbreviations: IQR = interquartile range; RP = radical prostatectomy; ISUP = International Society of Urological Pathology; PSA = prostate-specific antigen.

**Table 2:** Patient and clinicopathological characteristics of 86 radical prostatectomy patients between 03/2018 and 12/2019, stratified according to mid-term urinary continence status; all values are median (IQR) or frequencies (%).

	Overall, N = 68 (100%)	Continent, N = 55 (81%)	Incontinent, N = 13 (19%)	*p*-Value
Length of urethral sphincter, coronal, in mm Median (IQR)	14.7 (13.0, 16.7)	15.1 (13.8, 16.9)	12.5 (11.9, 14.2)	0.009
Length of urethral sphincter, sagital, in mm Median (IQR)	15.1 (12.8, 16.8)	15.4 (14.4, 17.4)	11.1 (9.8, 13.7)	<0.001
Length of urethral sphincter, axial, in mm Median (IQR)	10.2 (9.2, 11.2)	9.7 (9.1, 11.2)	10.5 (10.1, 11.1)	0.5
Diameter of urethral sphincter, coronal, in mm Median (IQR)	9.1 (7.9, 10.1)	9.1 (7.9, 10.1)	9.4 (8.9, 9.9)	0.5
Lee-type, n (%)				0.4
A	11 (16%)	8 (15%)	3 (23%)	
B	5 (7%)	3 (6%)	2 (15%)	
C	5 (7%)	4 (8%)	1 (7.7%)	
D	47 (69%)	40 (73%)	7 (54%)	
Age in years, Median (IQR)	66 (58, 72)	64 (58, 69)	72 (68, 74)	0.006
PSA, in ng/mL, Median (IQR)	7 (5, 10)	7 (5, 10)	7 (6, 11)	0.7
Body-Mass Index, Median (IQR)	25.6 (24.0, 27.2)	25.8 (24.0, 27.4)	24.6 (24.1, 27.1)	0.5
Prostate volume, in mL Median (IQR)	35 (28, 45)	35 (26, 45)	39 (30, 55)	0.5
D’Amico Score, n (%)				0.030
Low	5 (8%)	5 (9%)	0 (0%)	
Intermediate	39 (59%)	35 (65%)	4 (33%)	
High	22 (33%)	14 (26%)	8 (67%)	
ISUP Score at biopsy, n (%)				0.08
ISUP1	7 (10%)	6 (11%)	1 (8%)	
ISUP2	26 (38%)	24 (44%)	2 (15%)	
ISUP3	17 (25%)	14 (25%)	3 (23%)	
ISUP4	10 (15%)	7 (12%)	3 (23%)	
ISUP5	8 (12%)	4 (8%)	4 (31%)	
Pathological ISUP Score, n (%)				0.008
ISUP1	8 (12%)	6 (11%)	2 (18%)	
ISUP2	30 (46%)	27 (50%)	3 (27%)	
ISUP3	14 (22%)	14 (26%)	0 (0%)	
ISUP4	2 (3%)	1 (2%)	1 (9.1%)	
ISUP5	11 (17%)	6 (11%)	5 (45%)	
Nerve sparing approach, n (%)				0.019
None	6 (9%)	2 (4%)	4 (31%)	
Yes	62 (91%)	53 (96%)	9 (31%)	
pT-stage, n (%)				0.2
pT2	41 (60%)	35 (64%)	6 (46%)	
>pT2	27 (40%)	20 (36%)	7 (54%)	
pN-stage, n (%)				0.2
pN0	60 (88%)	50 (91%)	10 (77%)	
pN1	3 (4%)	2 (4%)	1 (8%)	
pNx	5 (8%)	3 (6%)	2 (15%)	
Surgical approach, n (%)				0.012
Robotic-assisted	37 (54%)	34 (62%)	3 (23%)	
Open	31 (46%)	21 (38%)	10 (77%)	

Abbreviations: IQR = interquartile range; RP = radical prostatectomy; ISUP = International Society of Urological Pathology; PSA = prostate-specific antigen.

**Table 3:** Univariable and multivariable logistic regression models predicting urinary continence at medium follow-up following radical prostatectomy, defined as none or one safety pad per 24 h.

	Univariable				Multivariable			
	Odds Ratio	CI 2.5 %	CI 97.5 %	*p*-Value	Odds Ratio	CI 2.5 %	CI 97.5 %	*p*-Value
Urethral sphincter length, coronal	1.42	1.09	1.96	0.02	-	-	-	-
Urethral sphincter length, sagital	1.69	1.31	2.33	>0.001	1.67	1.22	2.52	0.005
Urethral sphincter length, axial	0.94	0.62	1.43	0.77	-	-	-	-
Diameter of urethral sphincter, coronal	0.88	0.57	1.33	0.54	-	-	-	-
Lee-type								
A	*Ref.*				-	-	-	-
B	0.56	0.06	5.91	0.61	-	-	-	-
C	1.50	0.13	35.90	0.76	-	-	-	-
D	2.14	0.40	9.72	0.34	-	-	-	-
Age	0.88	0.79	0.97	0.02	0.87	0.73	0.99	0.04
Prostate volume	0.99	0.95	1.03	0.63	-	-	-	-
pT-stage								
pT2	*Ref.*				-	-	-	-
>pT2	0.49	0.14	1.67	0.25	-	-	-	-
Surgical approach								
Open RP					*Ref.*			
Robotic-assisted RP	5.40	1.46	26.17	0.02	3.03	0.38	34.13	0.31
Nerve sparing approach								
None	*Ref.*				*Ref*.			
Yes	11.78	2.01	94.71	0.01	0.79	0.01	45.67	0.90
Pathological ISUP								
1/2/3	*Ref.*				Ref.			
4/5	0.12	0.03	0.51	0.004	0.18	0.02	1.92	0.15

Abbreviations: RP = radical prostatectomy; CI = confidence interval; ISUP = International Society of Urological Pathology.

**Table 4:** Univariable and multivariable logistic regression models predicting urinary continence at medium follow-up following radical prostatectomy, defined as none or one safety pad per 24 h.

	Univariable				Multivariable			
	Odds Ratio	CI 2.5 %	CI 97.5 %	*p*-Value	Odds Ratio	CI 2.5 %	CI 97.5 %	*p*-Value
Urethral sphincter length, coronal	1.42	1.09	1.96	0.02	1.35	1.01	1.96	0.045
Urethral sphincter length, sagital	1.69	1.31	2.33	>0.001	-	-	-	-
Urethral sphincter length, axial	0.94	0.62	1.43	0.77	-	-	-	-
Diameter of urethral sphincter, coronal	0.88	0.57	1.33	0.54	-	-	-	-
Lee-type								
A	*Ref.*				-	-	-	-
B	0.56	0.06	5.91	0.61	-	-	-	-
C	1.50	0.13	35.90	0.76	-	-	-	-
D	2.14	0.40	9.72	0.34	-	-	-	-
Age	0.88	0.79	0.97	0.02	0.87	0.75	0.98	0.04
Prostate volume	0.99	0.95	1.03	0.63	-	-	-	-
pT-stage								
pT2	*Ref.*				-	-	-	-
>pT2	0.49	0.14	1.67	0.25	-	-	-	-
Surgical approach								
Open RP					*Ref.*			
Robotic-assisted RP	5.40	1.46	26.17	0.02	2.35	0.36	18.01	0.37
Nerve sparing approach								
None	*Ref.*				*Ref*.			
Yes	11.78	2.01	94.71	0.01	0.65	0.03	22.08	0.79
Pathological ISUP								
1/2/3	*Ref.*				*Ref.*			
4/5	0.12	0.03	0.51	0.004	0.22	0.02	1.83	0.16

Abbreviations: RP = radical prostatectomy; CI = confidence interval; ISUP = International Society of Urological Pathology.

## Data Availability

All datasets generated for this study are included in the manuscript. B.H. had full access to all the data in the study and takes responsibility for the integrity of the data and the accuracy of the data analysis.

## Supplementary Materials

The following supporting information can be downloaded at: https://www.mdpi.com/article/10.3390/diagnostics12030701/s1, Table S1: Univariable and multivariable logistic regression models predicting urinary continence at medium follow-up following radical prostatectomy, defined as none or one safety pad per 24 h.

## References

[1] Pompe R.S., Tian Z., Preisser F., Tennstedt P., Beyer B., Michl U., Graefen M., Huland H., Karakiewicz P.I., Tilki D. (2017). Short- and Long-term Functional Outcomes and Quality of Life after Radical Prostatectomy: Patient-reported Outcomes from a Tertiary High-volume Center. Eur. Urol. Focus.

[2] Theissen L., Preisser F., Wenzel M., Humke C., Roos F.C., Kluth L.A., Becker A., Banek S., Bodelle B., Köllermann J. (2019). Very Early Continence After Radical Prostatectomy and Its Influencing Factors. Front. Surg..

[3] Whiting P.F., Moore T.H., Jameson C.M., Davies P., Rowlands M.-A., Burke M., Beynon R., Savovic J., Donovan J.L. (2016). Symptomatic and quality-of-life outcomes after treatment for clinically localised prostate cancer: A systematic review. Br. J. Urol..

[4] Borges R.C., Tobias-Machado M., Gabriotti E.N., Figueiredo F.W.D.S., Bezerra C.A., Glina S. (2019). Post-radical prostatectomy urinary incontinence: Is there any discrepancy between medical reports and patients’ perceptions?. BMC Urol..

[5] Lee S.E., Byun S.-S., Lee H.J., Song S.H., Chang I.H., Kim Y.J., Gill M.C., Hong S.K. (2006). Impact of variations in prostatic apex shape on early recovery of urinary continence after radical retropubic prostatectomy. Urology.

[6] Paparel P., Akin O., Sandhu J.S., Otero J.R., Serio A.M., Scardino P.T., Hricak H., Guillonneau B. (2009). Recovery of Urinary Continence after Radical Prostatectomy: Association with Urethral Length and Urethral Fibrosis Measured by Preoperative and Postoperative Endorectal Magnetic Resonance Imaging. Eur. Urol..

[7] Marenco J., Orczyk C., Collins T., Moore C., Emberton M. (2019). Role of MRI in planning radical prostatectomy: What is the added value?. World J. Urol..

[8] Mungovan S.F., Sandhu J., Akin O., Smart N., Graham P., Patel M.I. (2017). Preoperative Membranous Urethral Length Measurement and Continence Recovery Following Radical Prostatectomy: A Systematic Review and Meta-analysis. Eur. Urol..

[9] Colarieti A., Thiruchelvam N., Barrett T. (2021). Evaluation of image-based prognostic parameters of post-prostatectomy urinary incontinence: A literature review. Int. J. Urol..

[10] Wenzel M., Preisser F., Mueller M., Theissen L.H., Welte M.N., Hoeh B., Humke C., Bernatz S., Bodelle B., Würnschimmel C. (2021). Effect of prostatic apex shape (Lee types) and urethral sphincter length in preoperative MRI on very early continence rates after radical prostatectomy. Int. Urol. Nephrol..

[11] Kitamura K., China T., Kanayama M., Nagata M., Isotani S., Wakumoto Y., Muto S., Ide H., Horie S. (2019). Significant association between urethral length measured by magnetic resonance imaging and urinary continence recovery after robot-assisted radical prostatectomy. Prostate Int..

[12] Song W., Kim C.K., Park B.K., Jeon H.G., Jeong B.C., Seo S.I., Jeon S.S., Choi H.Y., Lee H.M. (2017). Impact of preoperative and postoperative membranous urethral length measured by 3 Tesla magnetic resonance imaging on urinary continence recovery after robotic-assisted radical prostatectomy. Can. Urol. Assoc. J..

[13] Coakley F.V., Eberhardt S., Kattan M.W., Wei D.C., Scardino P.T., Hricak H. (2002). Urinary Continence After Radical Retropubic Prostatectomy: Relationship with Membranous Urethral Length on Preoperative Endorectal Magnetic Resonance Imaging. J. Urol..

[14] Basourakos S.P., Ramaswamy A., Yu M., Margolis D.J., Hu J.C. (2021). Racial Variation in Membranous Urethral Length and Postprostatectomy Urinary Function. Eur. Urol. Open Sci..

[15] Hu J.C., Ehdaie B., Sandhu J., Sjoberg D.D., Carlsson S.V., Tzeng M., Vickers A.J. (2020). Asian-American Race and Urinary Continence After Radical Prostatectomy. Eur. Urol. Open Sci..

[16] Preisser F., Theissen L., Wild P., Bartelt K., Kluth L., Köllermann J., Graefen M., Steuber T., Huland H., Tilki D. (2021). Implementation of Intraoperative Frozen Section During Radical Prostatectomy: Short-term Results from a German Tertiary-care Center. Eur. Urol. Focus.

[17] Barentsz J.O., Weinreb J.C., Verma S., Thoeny H.C., Tempany C.M., Shtern F., Padhani A., Margolis D., Macura K.J., Haider M.A. (2016). Synopsis of the PI-RADS v2 Guidelines for Multiparametric Prostate Magnetic Resonance Imaging and Recommendations for Use. Eur. Urol..

[18] RCT (2017). R: A Language and Environment for Statistical Computing. https://wwwr-projectorg2017.

[19] Kim L.H., Patel A., Kinsella N., Sharabiani M.T., Ap Dafydd D., Cahill D. (2020). Association Between Preoperative Magnetic Resonance Imaging–based Urethral Parameters and Continence Recovery Following Robot-assisted Radical Prostatectomy. Eur. Urol. Focus.

[20] Cho D.S., Choo S.H., Kim S.J., Shim K.H., Park S.G., Kim S.I. (2020). Postoperative membranous urethral length is the single most important surgical factor predicting recovery of postoperative urinary continence. Urol. Oncol. Semin. Orig. Investig..

[21] Satake Y., Kaiho Y., Saito H., Yamada T., Kawamorita N., Yamashita S., Mitsuzuka K., Yamada S., Ito A., Arai Y. (2018). Estimated Minimal Residual Membranous Urethral Length on Preoperative Magnetic Resonance Imaging Can Be a New Predictor for Continence After Radical Prostatectomy. Urology.

[22] Sauer M., Tennstedt P., Berliner C., Well L., Huland H., Budäus L., Adam G., Beyersdorff D. (2019). Predictors of Short and Long Term Urinary Incontinence after Radical Prostatectomy in Prostate MRI: Significance and Reliability of Standardized Measurements. Eur. J. Radiol..

[23] Matsushita K., Kent M.T., Vickers A., Von Bodman C., Bernstein M., Touijer K.A., Coleman J., Laudone V.T., Scardino P.T., Eastham J.A. (2015). Preoperative predictive model of recovery of urinary continence after radical prostatectomy. Br. J. Urol..

[24] Ahn H., Choi S.-K., Park S. (2015). Randomized clinical trial of a bladder neck plication stitch during robot-assisted radical prostatectomy. Asian J. Androl..

[25] Haese A., Knipper S., Isbarn H., Heinzer H., Tilki D., Salomon G., Michl U., Steuber T., Budäus L., Maurer T. (2019). A comparative study of robot-assisted and open radical prostatectomy in 10 790 men treated by highly trained surgeons for both procedures. Br. J. Urol..

[26] Hoeh B., Wenzel M., Hohenhorst L., Köllermann J., Graefen M., Haese A., Tilki D., Walz J., Kosiba M., Becker A. (2022). Anatomical Fundamentals and Current Surgical Knowledge of Prostate Anatomy Related to Functional and Oncological Outcomes for Robotic-Assisted Radical Prostatectomy. Front. Surg..

[27] Michl U., Tennstedt P., Feldmeier L., Mandel P., Oh S.J., Ahyai S., Budäus L., Chun F.K., Haese A., Heinzer H. (2016). Nerve-sparing Surgery Technique, Not the Preservation of the Neurovascular Bundles, Leads to Improved Long-term Continence Rates After Radical Prostatectomy. Eur. Urol..

