# Supplementary material for: Urethral Sphincter Length but Not Prostatic Apex Shape in Preoperative MRI Is Associated with Mid-Term Continence Rates after Radical Prostatectomy
Source: Diagnostics (Basel). 2022 Mar 13;12(3):701. doi: 10.3390/diagnostics12030701 (PMC8947169; doi:10.3390/diagnostics12030701)
Supplement: Supplementary file 1 [file diagnostics-12-00701-s001.zip › diagnostics-1603413-supplementary.pdf]

**Supplementary Table S1.** Univariable and multivariable logistic regression models predicting urinary continence at medium follow-up following radical prostatectomy, defined as none or one safety pad per 24hours.

| | Univariable | | | | Multivariable | | | |
|---|---|---|---|---|---|---|---|---|
| | **Odds ratio** | **CI 2.5 %** | **CI 97.5 %** | **p-value** | **Odds ratio** | **CI 2.5 %** | **CI 97.5 %** | **p-value** |
| **Urethral sphincter length, coronal** | 1.42 | 1.09 | 1.96 | 0.02 | - | - | - | - |
| **Urethral sphincter length, sagital** | 1.69 | 1.31 | 2.33 | >0.001 | - | - | - | - |
| **Urethral sphincter length, axial** | 0.94 | 0.62 | 1.43 | 0.77 | - | - | - | - |
| **Diameter of urethral sphincter, coronal** | 0.88 | 0.57 | 1.33 | 0.54 | - | - | - | - |
| **Lee-type** | | | | | | | | |
| A | *Ref.* | | | | *Ref.* | | | |
| B | 0.56 | 0.06 | 5.91 | 0.61 | 7.49 | 0.25 | 288.47 | 0.26 |
| C | 1.50 | 0.13 | 35.90 | 0.76 | 0.06 | 0.01 | 2.78 | 0.14 |
| D | 2.14 | 0.40 | 9.72 | 0.34 | 4.04 | 0.54 | 35.08 | 0.17 |
| **Age** | 0.88 | 0.79 | 0.97 | 0.02 | 0.80 | 0.65 | 47.68 | 0.02 |
| **Prostate volume** | 0.99 | 0.95 | 1.03 | 0.63 | - | - | - | - |
| **pT-stage** | | | | | | | | |
| pT2 | *Ref.* | | | | - | - | - | - |
| >pT2 | 0.49 | 0.14 | 1.67 | 0.25 | - | - | - | - |
| **Surgical approach** | | | | | | | | |
| Open RP | | | | | *Ref.* | | | |
| Robotic-assisted RP | 5.40 | 1.46 | 26.17 | 0.02 | 3.16 | 0.38 | 29.90 | 0.28 |
| **Nerve sparing approach** | | | | | | | | |
| None | *Ref.* | | | | *Ref.* | | | |
| Yes | 11.78 | 2.01 | 94.71 | 0.01 | 1.16 | 0.05 | 47.68 | 0.92 |
| **Pathological ISUP** | | | | | | | | |
| 1/2/3 | *Ref.* | | | | *Ref.* | | | |
| 4/5 | 0.12 | 0.03 | 0.51 | 0.004 | 0.16 | 0.01 | 1.48 | 0.12 |

Abbreviations: RP= Radical prostatectomy; CI= Confidence interval; ISUP= International Society of Urological Pathology;
